# Cervical proprioception and its relationship with neck pain intensity in subjects with cervical spondylosis

**DOI:** 10.1186/s12891-019-2846-z

**Published:** 2019-10-15

**Authors:** Ravi Shankar Reddy, Jaya Shanker Tedla, Snehil Dixit, Mohammed Abohashrh

**Affiliations:** 1 Department of Medical Rehabilitation (Physical Therapy), College of Applied Medical Sciences, King Khalid University, Abha, 61421 Saudi Arabia; 2 Department of Basic Medical Sciences, College of applied medical sciences, King Khalid University, Abha, Saudi Arabia

**Keywords:** Neck pain, Cervical spondylosis, Proprioception, Pain intensity

## Abstract

**Background:**

Cervical proprioception is critical in the maintenance of posture and movements, so its assessment in different cervical conditions has gained importance in recent clinical practice. Studies reporting this assessment in subjects with cervical spondylosis (CS) have not previously been investigated. The goals of the study are (1) comparison of joint position error (JPE) in subjects with CS to healthy control group. (2) Correlation of neck pain intensity to cervical proprioception in patients with CS.

**Methods:**

In a Cross-sectional study, 132 subjects with CS and 132 healthy age-matched control subjects were evaluated for cervical JPE with the cervical range of motion device. The subjects were blindfolded and repositioned their heads to a target position, which was determined by the examiner previously and their repositioning accuracy (absolute error in degrees) was measured in the frontal (flexion and extension) and transverse planes (left rotation and right rotation). The CS subjects resting neck pain intensity was assessed using visual analog scale (VAS).

**Results:**

CS subjects showed statistically significantly larger JPEs compared to healthy control subjects in all the directions tested (flexion - 95% CI = 2.38–3.55, *p* < 0.001, extension - 95% CI =3.26–4.33, *p* < 0.001, left rotation - 95% CI = 2.64 - 3.83, *p* < 0.001, right rotation − 95% CI = 3.77–4.76, *p* < 0.001). The mean JPE errors in the CS group ranged from 6.27° to 8.28° and in the control group ranged from 2.36° to 4.48°. Pearson’s correlation coefficient showed a significant and positive relationship between neck pain intensity and cervical proprioception (*p* ≤ 0.001).

**Conclusions:**

Proprioception is impaired in subjects with CS when compared to healthy control group. Higher pain intensity was associated with greater cervical JPE in patients with CS.

## Background

Cervical spondylosis (CS) is an age-related chronic degenerative condition of the cervical spine with a prevalence rate of 3.3 patients per 1000 people in the general population [1, 2]. The usual occurrence of CS is at C5-C6 and C6-C7 levels, although higher levels may also be involved [3]. Even though age-related degeneration is the primary cause of degeneration, in younger patients injuries to the cervical disc can affect this degenerative process. Subjects with CS usually present with complaints of pain, tingling, numbness and weakness in the upper extremity, which will lead to significant disability and functional limitations [3].

Proprioception is a sense of bodily movement position, which includes position sense (joint position sense) and movement sense (kinesthesia) [4]. The ascending proprioceptive information reaches the central nervous system via the afferent pathway contributing to movement and postural neuromuscular control [4]. The cervical muscles have abundant muscle spindle density that reflects a rich proprioceptive system, which contributes to enhanced sensorimotor function and thereby play an important role in maintaining static and dynamic postures with effective motor control [5].

Studies have shown that cervical position sense is vital in maintaining joint stability under static and dynamic conditions and the development of clinical pain is predisposed by impaired proprioception [6]. Cervical proprioception is quantified by joint position error (JPE) measured in degrees. In CS, if the non-specific nature of problems is paired with impaired cervical proprioception, it is more likely that position sensibility is primarily affected by impairment in cervical muscles, joints, or capsules and, secondarily, by alteration in afferent proprioceptive tuning and integration [7, 8]. In CS, there may be impaired mechanoreceptor’s feedback, which might contribute to cervical muscle atrophy and joint degenerative changes resulting in unpredictable “giving away.” [8, 9]

An impaired position sense disturbs both neuronal and muscular control of the normal cervical joint function, which may result in the untimely production of unbalanced muscle force and place the joint at risk of trauma [8, 9].

Cervical pain can originate from a local spot or can spread to distant areas and develop into chronic syndrome in subjects with CS [8, 9]. Acute pain transforming into chronic pain is a complex process and not fully understood by researchers, and thereby the intensity of pain is a focus of research on subjects with neck pain [9–11]. Lee et al. conducted a study to show if there is any association between temporal aspects of pain (Pain frequency, duration, intensity) and cervical position sense in subjects with subclinical neck pain [6]. The neck pain intensity did not show consistent effect with cervical proprioceptive ability. Lee did not see consistent association may be because he sub grouped subjects as mild, moderate and severe pain intensity groups and compared the proprioception errors rather than seeing association between each subject pain intensity to his proprioceptive ability. To date, there are no studies that showed a consistent relationship between neck pain intensity and cervical proprioception, possibly because neck pain intensity is not easily quantified due to its subjective nature, particularly if neck pain is subclinical or occasional. Another approach is to evaluate the temporal aspect of pain intensity and its influence on neck proprioception in CS subjects. Therefore, the purpose of the study is to see the comparison of joint position error in subjects with CS to healthy control group and to see a relationship between neck pain intensity and cervical proprioception in patients with CS. The hypothesis of the study is that 1) cervical JPE will be greater in CS group compared to healthy control group. 2) Higher cervical pain intensity will be associated with greater cervical JPE in subjects with CS.

## Methods

Our study is a cross-sectional analysis and the subjects in this study’s sample were patients aged 30 to 60 years experiencing neck pain due to CS and referred from the orthopedic or neuro-medicine clinic to physical therapy. CS is defined as “neck pain subjects with the radiological findings confirming cervical degeneration (degenerative changes in the intervertebral discs, osteophytosis of the vertebral bodies, hypertrophy of the facets and laminal arches, and ligamentous and segmental instability) and cervical range is limited when compared with age-matched healthy subjects.” Subjects were included if the neck pain was the main presenting complaint, and if neck movements reproduced neck pain. Subjects with a history of neurologic disease, whiplash injury, cervical myelopathy, any inflammatory arthritis, tumors, infection involving the cervical spine, and vertebrobasilar artery insufficiency were excluded. For the age-matched healthy control subjects to be considered for inclusion, the subjects must have age-matched normal cervical spine ROM in all the planes, with no history of cervical spine injury, dizziness, or vertigo, or any other musculoskeletal complaints. All the subjects included in the study signed a consent form and the King Khalid University Ethics Committee approved the study (REC # 2016-01-06).

### Cervical JPE testing

**Fig. 1:**
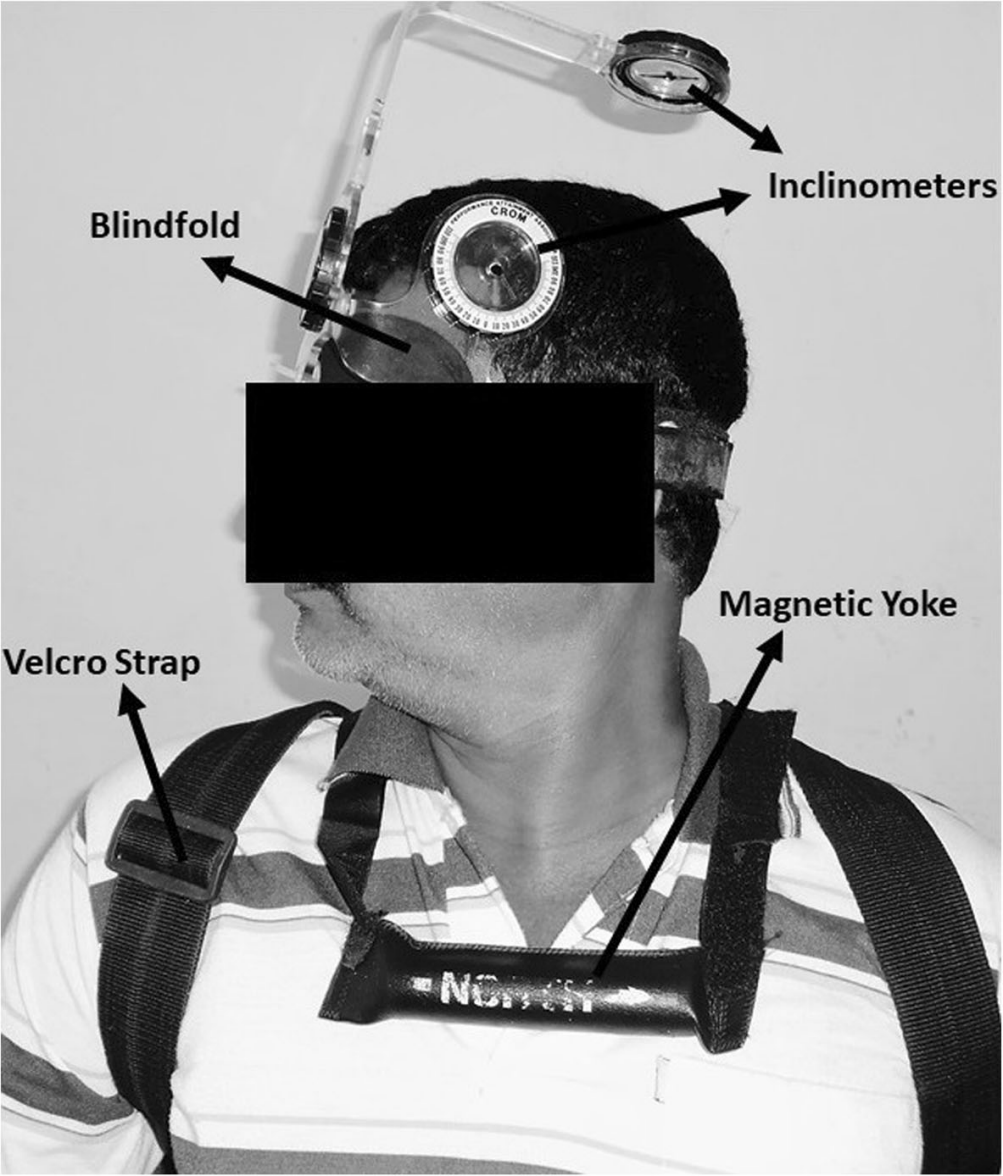
Procedure to evaluate cervical joint position error

**Fig. 2:**
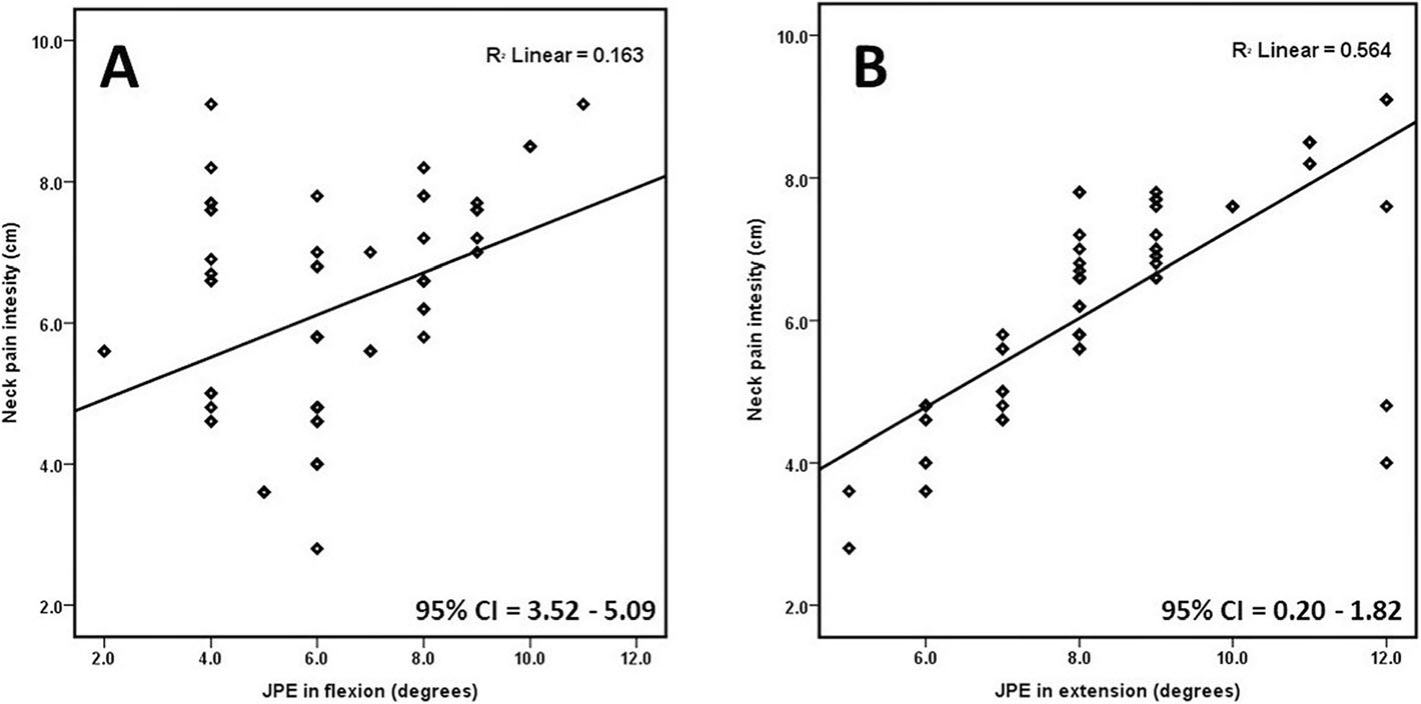
Relationship between neck pain intensity and **a** JPE in flexion (^0^), **b** JPE in extension (^0^)

The testing procedures implemented in this study are those adopted by Lee et al. [6]. The subjects sat upright in the chair with back straight, head facing straight ahead, and feet touching the ground, and this position was selected as the neutral head position. The study procedure was explained to all the subjects and standard instructions were given during their testing phase. A travel eye mask was used to blindfold the subject and a Velcro strap was used to fix and limit trunk and shoulder movement during the JPE testing procedure (Figs. 1 and 2).

The examiner secured the cervical range of motion (CROM) unit on the subject’s head with the Velcro strap and the magnetic yoke of the CROM device was arranged on the subject’s shoulder with arrow mark placed to the north. With the above position maintained, the examiner calibrated the CROM device to a neutral position. CROM is a valid device to measure cervical ROM and intratester reliability ranged from 0.62 to 0.91 and inter-tester reliability ranged from 0.74 to 0.87 [12]. To evaluate the JPE, the examiner held the subject’s head and moved slowly to the target head position, which is 50% of the maximum CROM (which was previously recorded by the examiner) [13] and held there for a period of 3 s. The subjects then memorized that target position and then the examiner slowly brought the subject’s head back to the neutral position. The subjects then were advised to reach the target position actively by moving the head and when subject reached the target position the reposition accuracy error was determined in degrees. The examiner evaluated JPE tests in sagittal (flexion, extension) and transverse plane (right and left rotation) directions. A simple chit method was adopted to randomize this order of testing. Each direction of JPE testing was performed three times and the mean error of these trials was used in the analysis. A single investigator administered all tests and no feedback was provided to the subjects during the testing procedure. The JPE tests were then compared between CS and healthy control subjects.

### Neck pain assessment

The CS subjects resting neck pain intensity was assessed using visual analog scale (VAS). The VAS is a psychometric response scale. When responding to a VAS item, patients make marks along the 100 mm line at the point they feel represents their current pain state. The VAS has been used extensively as an outcome measure. It has high test-retest reliability and validity [14].

### Statistical analysis

SPSS version 20.0 (IBM-SPSS Inc., Armonk, NY) was used to perform data analysis. All statistical values with *p* ≤ 0.05 were considered significant. All the values are represented as mean ± SD.

A Shapiro–Wilk test showed that the study data were normally distributed so an independent t test was used to compare cervical JPE differences between CS and healthy control subjects. Minimal detectable change (MDC) was computed, which is a statistical estimation of the minimum quantity of change that can be identified by a measure that matches to a noticeable true change in capacity versus a false or error change. MDC was computed by the formula: (Standard Error Mean (SEM) × 1.65 × √2), where SEM = standard deviation of the sample mean [15]. Pearson’s correlation coefficient (r) was computed to investigate the linear relationship between neck pain intensity and cervical JPE. We considered Pearson’s *r* > 0.75 = strong, r between 0.45 and 0.75 = moderate, and r below 0.45 = weak correlation [16].

## Results

In this study, the sample size was 264 people (132 patients in each group). CS and healthy control groups showed no significant statistical differences for age, gender, and body mass index (BMI) (*p* > 0.05) (Table 1) at baseline. CS subjects showed significantly larger JPE than healthy control subjects in all the directions tested (flexion, extension, right and left rotation) with a *p*-value < 0.001 (Table 2).

**Table 1:** Demographic characteristics of the study population

Variables	Cervical spondylosis Group	Control Group	*p* value
Age (years)	47.18 ± 6.54	45.07 ± 8.02	0.100
Gender (n)	96:36	100:32	0.693
BMI (kg/m^2^)	24.30 ± 2.99	24.24 ± 2.21	0.458
Neck pain intensity (cm)	6.21 ± 1.50	0	< 0.001
NDI score (%)	31.81 ± 10.66	0	< 0.001

*BMI* body mass index, *NDI* neck disability index

**Table 2:** Comparison of JPE between cervical spondylosis and control group

Variables	Cervical Spondylosis group (*n* = 132) (Mean ± SD)	Healthy control group (*n* = 132) (Mean ± SD)	Actual Difference between CS and Control group	95% CI		SEM	MDC	*p* value
				Lower	Upper			
JPE in flexion (^0^)	6.33 ± 2.02	3.36 ± 1.27	2.97	2.38	3.55	0.19	0.44	< 0.001
JPE in extension (^0^)	8.28 ± 1.80	4.48 ± 1.26	3.80	3.26	4.33	0.21	0.48	< 0.001
JPE in left rotation (^0^)	6.27 ± 1.96	3.03 ± 1.45	3.24	2.64	3.83	0.20	0.46	< 0.001
JPE in right rotation (^0^)	6.63 ± 1.75	2.36 ± 1.03	4.27	3.77	4.76	0.22	0.51	< 0.001

*JPE* joint position error, *CI* confidence interval, *SEM* standard error of the mean, *MDC* minimal detectable change

Comparing all the movement directions in the CS and healthy control groups, the JPEs were largest in cervical extension (CS groups = 8.28 ± 1.80°; healthy group = 4.48 ± 1.26°) with SEM of 0.21° and minimal detectable change (MDC) of 0.48°. The smallest JPE was seen for rotation left in the CS group (6.27 ± 1.96°) with SEM of 0.20°, MDC of 0.46° and right rotation in the healthy control group (2.36 ± 1.03°) with SEM of 0.22°, MDC of 0.51° (Table 2).

**Fig. 3:**
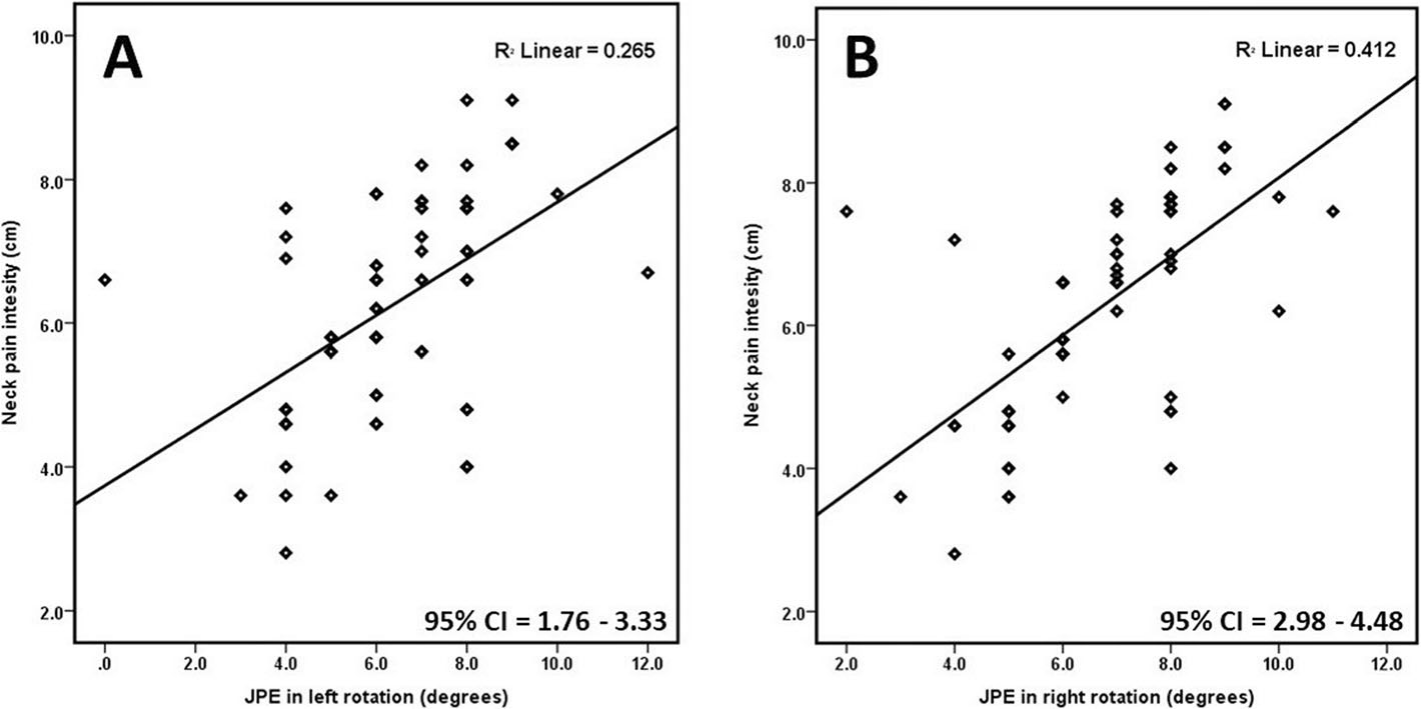
Relationship between neck pain intensity and **a** JPE in left rotation (^0^), **b** JPE in right rotation (^0^)

Pearson’s correlation coefficient (r) showed a positive and significant relation between neck pain intensity and JPEs in all directions tested as summarized in Fig. 3 and Table 3. A strong positive correlation was seen between neck pain intensity and JPE in left rotation with Pearson’s *r* = 0.78; CI = 1.76–3.33; *p* < 0.001 and moderate positive correlation was observed between neck pain intensity and JPE in flexion (*r* = 0.67; CI = 3.52–5.09; *p* = 0.001), extension (*r* = 0.59; CI = 0.20–1.82; *p* < 0.001), and right rotation (*r* = 0.66; CI = 2.98–4.48; *p* < 0.001).

**Table 3:** Coefficient of correlation between pain intensity and joint position errors in CS subjects

		JPE in Flexion (^0^)	JPE in extension (^0^)	JPE in left Rotation (^0^)	JPE in right rotation (^0^)
Neck pain intensity (cm)	r	0.67	0.59	0.78	0.66
95% CI	Lower	3.52	0.20	2.98	1.76
	Upper	5.09	1.82	4.48	3.33
	p	0.001	< 0.001	< 0.001	< 0.001

*CS* cervical spondylosis, *JPE* joint position error, *CI* confidence interval

## Discussion

This study demonstrated that cervical proprioceptive errors (JPEs) are significantly larger in the CS group than in the healthy control group indicating that cervical proprioception is impaired in subjects with CS. Cervical proprioceptive errors in the CS group significantly and positively correlated with neck pain intensity. The cervical JPE increased with increased neck pain intensity showing that an increase in pain intensity will impair the proprioceptive functionality.

This study finding, which showed substantial proprioceptive impairment in terms of increased JPE in the CS group, is in accordance with findings from previously published results that involved subjects with other types of neck conditions [6, 9, 17, 18]. Rix et al.’s study showed a mean JPE of 6.3° in flexion, 5.2° in extension, 6.9° in right rotation, and 4.2° in left rotation in subjects with chronic, non-traumatic cervical spine pain [18]. In Roren et al., the study demonstrated an absolute error of 3.6° to 3.7° for healthy subjects and 6.1° to 6.3° for neck disorder subjects when a subject’s head reposition was measured with an ultrasound-based technique [19]. Wibault et al. conducted a study on subjects with cervical radicular symptoms caused by disc prolapse and showed a smaller mean JPE of 3.8° in right rotation and 2.7° in left rotation [20] compared with our study results (right rotation = 6.63°, left rotation = 6.27°). These may be due to the differences in methodological considerations in both studies. Caution should be exercised when comparing our study results for cervical JPE with studies previously published because of differences in study methods, evaluations, and interpretations used.

The reasons for the impaired proprioception in the CS group may be due to the following causes. In CS there may be altered proprioceptive afferent signals from the skin, joints, and muscle spindle receptors due to degenerative changes to capsuloligamentous structures and mechanoreceptors [21] as well as muscle dysfunction [22]. The decrease in cervical muscle strength has been established in neck pain syndromes like CS [23–25]. The reported decrease in cervical muscle strength compared with healthy individuals varies by about 20 to 90% [23, 25–27]. Patients with CS have been shown to exhibit greater fatigue and weakness of both deep flexor and dorsal neck extensor muscles at high force levels compared with healthy subjects at electromyography [28]. Decreased muscle strength and increased muscle fatigue can alter the firing of sensory receptors (Golgi tendon organ or muscle spindles) and thereby influence afferent inputs resulting in altered proprioception [29, 30].

To date, to our knowledge, this is the first study of its kind that has evaluated and shown a significant positive correlation between neck pain intensity and position sense in subjects with CS. Pain models that were experimentally induced showed a positive influence between pain and proprioception [31]. The substances that are chemically mediated during the pain response might alter free nerve ending discharges due to sensitization and produce abnormal pain afferents (gamma-motor neuron and muscle spindle), thus impairing kinesthetic input [32]. Contrary to our study, Lee et al.’s study did not show any association between neck pain intensity and cervical proprioceptive sense in chronic neck pain subjects; rather, they noted that a higher pain frequency was positively associated with increased JPEs [6]. Our study results cannot be compared with et al. study as the sample characteristics and cervical proprioception testing methodological considerations are different from the current study. Further studies are required to see the association between neck pain frequency, duration, and intensity and proprioception in a larger sample of CS subjects.

To measure cervical proprioception, this study implemented the active head repositioning to the target method, which was previously used by several authors in clinical settings [13, 33, 34] and was found to be a reliable method. The number of testing trials or movement repetitions in each direction was limited to three to minimize the effect of fatigue of cervical muscles on JPE. Different authors recommended a greater number of trials in each testing direction to improve the reliability of position sense measurement [35], but increasing the number of repetitions can possibly lead to increased pain and fatigability, which may alter the test results of JPEs in subjects with CS.

### Limitations

The self-assessment of neck pain intensity on a VAS by the subject may be influenced by many factors like beliefs, socio-economic status or psychological status as pain is a subjective feeling and there by influencing the study results. The present study recorded absolute errors only. Variable and constant errors were not observed or else direction and magnitude of JPEs have been recorded, which might have given more meaningful information regarding the direction of JPEs tested. All the subjects were assessed for cervical JPE only after a single practice session may be results would be different if more practice sessions were provided to the subjects prior to the actual testing.

## Conclusion

CS subjects showed significantly greater cervical JPEs than healthy controls in all the movement directions tested. Hence, this study proved proprioception is impaired in subjects with CS, and a higher pain intensity was associated with greater cervical JPE.

## Data Availability

All data are available at the medical rehabilitation department (College of Applied Medical Sciences) on application to the primary author Ravi Shankar Reddy (rshankar@kku.edu.sa).

## Abbreviations

CROM device: Cervical range of motion device
CS: Cervical spondylosis
JPE: Joint position error
